# Polydopamine grafting polyether ether ketone to stabilize growth factor for efficient osteonecrosis repair

**DOI:** 10.1038/s41598-025-86965-1

**Published:** 2025-01-29

**Authors:** Yi Sun, Jingyun Liu, Kaijia Chen, Nannan Zhong, Chengpeng He, Xinming Luan, Xiaobei Zang, Jianbo Sun, Ning Cao, Wenbo Wang, Qiang Ren

**Affiliations:** 1 Department of Bone Joint, Binzhou Medical University Hospital, No. 661 Huanghe 2nd Road, Binzhou, 256600 China; 2 Department of Orthopedics, Liaocheng People’s Hospital, No. 67, Dongchang West Road, Liaocheng, 252000 China; 3 School of Materials and Engineering, China University of Petroleum (East China), No.66, West Changjiang Road, Huangdao District, Qingdao, 266580 China; 4 Department of Orthopedics, The Second Affiliated Hospital of Shandong, University of Traditional Chinese Medicine, Jinan, 250001 China

**Keywords:** Polyether ether ketone (PEEK), Surface modification, Bone forming peptide (BFP), Vascular endothelial growth factor (VEGF), Osteonecrosis, Chemical modification, Medical research

## Abstract

This study examines the biocompatibility, osteogenic potential, and effectiveness of polyether ether ketone (PEEK) composites for treating osteonecrosis, seeking to establish a theoretical basis for clinical application. A range of PEEK composite materials, including sulfonated polyether ether ketone (SPEEK), polydopamine-sulfonated polyether ether ketone (SPEEK-PDA), bone-forming peptide-poly-dopamine-sulfonated polyether ether ketone (SPEEK-PDA-BFP), and vascular endothelial growth factor-poly-dopamine-sulfonated polyether ether ketone (SPEEK-PDA-VEGF), were constructed by concentrated sulfuric acid sulfonation, polydopamine modification and grafting of bioactive factors. The experiments involved adult male New Zealand rabbits aged 24–28 weeks and weighing 2.6–4 kg. The SPEEK-PDA-BFP possesses the smallest water contact angle, indicating the highest hydrophilicity, with its surface characterized by a rich density of clustered BFP particles. The SPEEK-PDA-BFP exhibits superior adhesion, proliferation, and differentiation capabilities, along with pronounced bacteriostatic effects, which are attributed to its dense particle clusters. The SPEEK-PDA-BFP facilitates the formation of regular and dense bone trabeculae. Comparative study on treating osteonecrosis with SPEEK-PDA-VEGF and SPEEK-PDA-BFP highlighted the superior formation of mature bone trabeculae and angiogenic protein CD31 around SPEEK-PDA-VEGF. The PEEK composite materials have good biocompatibility, osteogenic activity and bone repair activity. In particular, SPEEK-PDA-VEGF composite materials have the best effect on bone repair.

## Introduction

Osteonecrosis is a common clinical disease caused by reduced blood supply to bone tissue, mainly affecting large joints such as hips and knees^1^. Its pathogenic factors are extensive, including trauma, long-term alcoholism, hormone abuse, hyperlipidemia, decompression sickness, and hematological diseases^2–4^. It is generally believed that the earlier the diagnosis of osteonecrosis, the better the treatment effect. Therefore, scholars are increasingly paying attention to early treatment before articular surface collapse^5,6^. At present, surgical treatment is the main way to treat osteonecrosis in the early clinical stage, in which core decompression combined with bone transplantation is the most mainstream surgical method. Bone graft is a key part of bone transplantation surgery, and the integration of implant materials and surrounding bone tissue has a decisive impact on the therapeutic effect of bone tissue regeneration and repair^7,8^. At present, orthopedic implant materials widely used in clinics mainly include metal materials, ceramics, and polymer materials. However, in long-term clinical application, it has been found that the elastic modulus of metal and ceramic is much higher than that of human bone tissue, and a stress shielding effect will be generated when stressed, which may lead to loosening or even fracture^9^. The metal particles released by long-term wear of metal implants can cause inflammation in the surrounding tissues, and eventually loosen and fall off the implants^10^. Polymer materials have emerged as excellent grafts in recent years, which can be processed to adjust their mechanical properties and biological activities, and have great potential for bone integration, and are more and more widely used in clinical implantation surgery.

Polyether ether ketone (PEEK) is a polymer formed by interconnecting ketone and ether bonds that occur repeatedly in the backbone structure. It has good biocompatibility and excellent mechanical properties and is an ideal bone graft^11,12^. However, the bone integration and antibacterial properties of PEEK materials are poor, which is not conducive to cell adhesion and proliferation and makes it easy to infect bacteria. Surface modification is the main method to improve the biological activity and antibacterial properties of PEEK materials, including physical modification, chemical modification, surface layer modification, and composite modification^13,14^. Among these methods, composite modification by incorporating sulfonated polyether ether ketone (SPEEK) offers new opportunities for enhancing structural properties and functional performance of PEEK-based materials. Composite materials based on SPEEK have been researched and applied in various fields. A type of hybrid membrane based on SPEEK demonstrates high mechanical strength and oxidative stability in high temperature and low humidity fuel cells application^15^. This highlights the potential of SPEEK-based composites to overcome the limitations of traditional physical and chemical modification methods, particularly in creating uniform and stable surface coatings. Additionally, a sulfonated-fluorinated copolymer blending membrane containing SPEEK has been designed for use as the electrolyte in polymer electrolyte fuel cells, showing superior performance and stability^16^. Therefore, by optimizing the composition and manufacturing techniques of SPEEK-based composites, satisfactory surface characteristics can be developed to support cell adhesion and proliferation preferably, promoting the use of PEEK materials in bone grafts and other biomedical fields. The sulfonation of concentrated sulfuric acid can form a uniform three-dimensional pore structure on the surface of the material and provide a site for the subsequent grafting reaction of the material. Studies have shown^17,18^ that the pore network structure of the material is conducive to the growth of osteoblasts and the transport of nutrients, and promotes the repair of osteonecrosis. Although the biological activity of sulfonated PEEK material was improved, it still could not achieve the ideal bone integration. In this regard, the researchers combined the transplanted material with the bioactive material by a crosslinking agent to make up for the shortcomings of the insufficient biological activity of the material. Among them, the dopamine crosslinker has the advantages of high efficiency, safety, and simplicity, and can significantly improve the biocompatibility of the material. Dopamine (DA) is a neurotransmitter secreted by mammals that polymerizes in a weakly alkaline solution to form polydopamine (PDA). The amino functional groups on the surface of the PDA can be combined with the substrate to form a dense PDA film on the surface of the material. In addition, the phenol hydroxyl group on the surface of PDA can achieve secondary modification of the material by binding metal ions, proteins, and other active substances, further improving the surface state and properties of the material^19,20^. Although the biological activity of PEEK material combined with PDA was improved, it was not enough to achieve bone repair in osteonecrotic areas. Since the formation of new bone and new blood vessels is a key factor in promoting the repair of osteonecrosis, osteogenic factors and angiogenic factors can be grafted onto the surface of PEEK material to improve the efficiency of bone repair. Bone morphogenetic protein (BMP), a member of the transforming growth factor β superfamily, is one of the most effective bone-inducing proteins currently known, which can significantly promote the proliferation, differentiation, and cell activity of osteoblasts^21,22^. Studies have found^23,24^ that bone-forming peptide (BFP), as a polypeptide fragment extracted from BMP, can more significantly promote the proliferation and differentiation of osteoblasts. Vascular endothelial growth factor (VEGF) also plays an important role in bone repair. VEGF can bind to the vascular endothelial growth factor receptor, migrate and aggregate bone marrow mesenchymal stem cells and osteoblasts to the repair site, promote the proliferation and differentiation of osteoblasts, and directly participate in the regeneration and repair of bone tissue^25–27^. At the same time, VEGF can bind to tyrosine kinase receptors to promote the proliferation and differentiation of vascular endothelial cells and induce angiogenesis at the site of osteonecrosis^28^. Therefore, compared with BFP, which only has osteogenic activity, VEGF with both osteogenic and angiogenic activity can significantly improve the bone integration of PEEK material, effectively improve the blood supply disorder at the site of osteonecrosis, and reverse the pathological process of osteonecrosis^29^.

The research group prepared PEEK material with a three-dimensional pore structure by sulfonation of concentrated sulfuric acid and then grafted VEGF or BFP onto the surface of the material by PDA to achieve the surface modification of PEEK material. The bone integration and bone repair ability of different PEEK composites were determined through the preparation evaluation experiment, in vitro biological performance experiment, and in vivo bone repair experiment, and the effects of two different bioactive substances, BFP and VEGF, on the bone integration of PEEK composites were compared.

## Materials and methods

### Materials and instruments

PEEK composite materials: In vitro test material is a 10 mm diameter, 2 mm thick disc, in vivo test material is 5 mm diameter, length of 15 mm cylindrical rod body.

Main reagents: α-MEM culture-medium (China meilunbio), fetal bovine serum (China meilunbio), PBS buffer solution (China meilunbio), penicillin/streptomycin (China meilunbio), CCK-8 test kit (China meilunbio), basic phosphateEnzyme detection kit (China meilunbio), CD31 antibody (USA santaCruz), OCN antibody (USA santaCruz), all secondary antibodies (USA abbkine).

Experimental Cells: MC3T3-E1 cells.

Experimental Strains: E. coli; Staphylococcus aureus (ATCC25923).

Experimental animals: Adult male New Zealand rabbits, aged 24–28 weeks and weighing 2.6–4.0 kg. Four adult male New Zealand rabbits were used as blank controls, and the remaining 36 rabbits were studied in vivo. Animal feeding, surgical operation, and specimen collection were all conducted in the animal laboratory of Binzhou Medical University Hospital. Animal experiments were conducted in strict compliance with animal ethical requirements and were all approved by the Experimental Animal Ethics Committee (approval number: 20211008-91). All experiments were performed in accordance with relevant guidelines and regulations. The study is reported in accordance with ARRIVE guidelines.

Experimental instruments: CO2 cell incubator (ThermoFisherScientific, USA), general-purpose table centrifuge (Eppendorf, Germany), multifunctional enzyme labeling instrument (MolecuLarDevices, USA), high power CNC ultrasonic cleaner (Ningbo Xinzhi Biotechnology Co., LTD.), contact angle measuring instrument (Shanghai Zhongchen Digital Technology Equipment Co., LTD.), X-ray photoelectron spectroscopy analyzer (Thermo Fisher Technology Co., LTD.), Fourier attenuated total reflection infrared spectroscopy (Thermo Fisher Technology Co., LTD.), scanning electron microscope (Japan Hitachi), universal material mechanics testing machine (Shanghai Fenji Instrument Co., LTD.), Micro-CT (Germany Bruker Co., LTD.).

### Preparation of SPEEK-PDA-BFP and SPEEK-PDA-VEGF

**Fig. 1:**
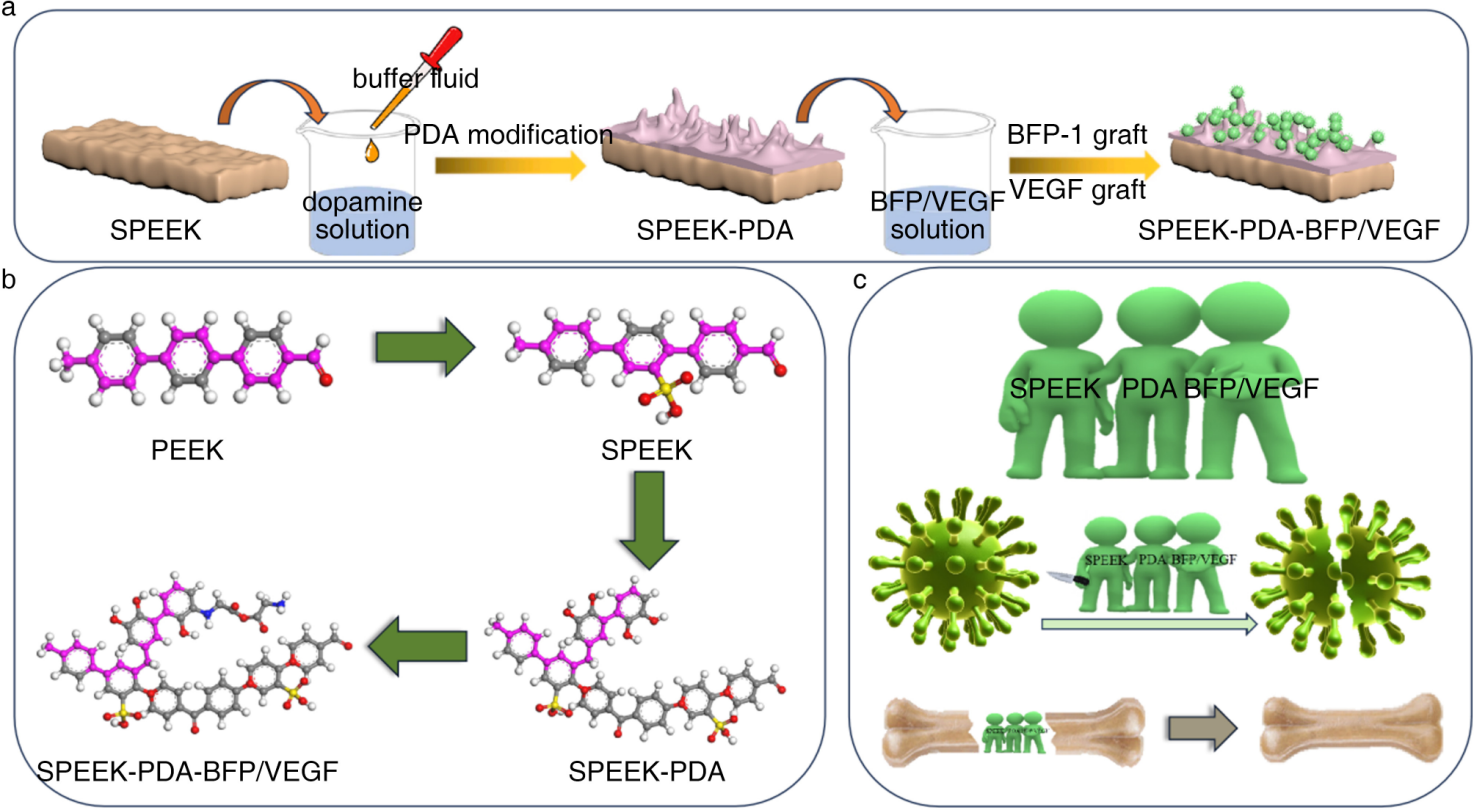
(a) Preparation process of PEEK composite materials.(b) Molecular structure of PEEK composite materials.(c) Mechanism of PEEK composites in treating osteonecrosis.

PEEK samples were processed into 20 mm×20 mm×1 mm round sheets, and the samples were polished with silicon sandpaper. The polished PEEK samples were cleaned by ultrasonic shock with acetone, alcohol, and deionized water successively for 30 min to remove surface impurities. The cleaned sample was put into the oven at 60℃ to dry. The sample was immersed in 98% concentrated sulfuric acid for 10 min to obtain sulfonated PEEK (SPEEK). The SPEEK samples were cleaned by ultrasonic shock with deionized water, acetone, alcohol, and deionized water respectively for 30 min to remove the remaining sulfur-containing groups. After sulfonation, SPEEK was modified by polydopamine. The sulfonated PEEK sample was placed into 2 kg·m^−3^ dopamine solution and a small amount of Tris-HCl buffer solution was added (10 mol·m^−3^, pH = 8.5). PDA-modified SPEEK (SPEEK-PDA) was obtained by stirring at room temperature for 24 h. The SPEEK-PDA sample is repeatedly rinsed in deionized water to remove the PDA remaining on the surface due to physical adsorption. VEGF and BFP were dissolved in a phosphate buffer solution (PBS), and the concentration of VEGF and BFP was 1 kg·m^−3^. SPEEK-PDA samples were immersed in VEGF or BFP solution respectively, and grafted with VEGF and BFP at 4℃. After 24 h, the samples were removed and repeatedly rinsed with deionized water to obtain VEGF-modified SPEEK-PDA (SPEEK-PDA-VEGF) and BFP-modified SPEEK-PDA (SPEEK-PDA-BFP). See Fig. 1 for an introduction.

### Preparation and evaluation experiment of PEEK composite materials

The preparation and evaluation experiment of PEEK composites includes the detection of chemical composition, surface wettability, and micro-morphology of the material. The chemical composition of the material was detected by Fourier transform attenuated total reflection infrared spectroscopy (FTIR) and X-ray photoelectron spectroscopy (XPS). FTIR identifies the chemical composition by detecting the change of groups and chemical bonds on the surface of the material and identifies whether new chemical bonds are generated on the surface of the material by different surface functional group absorption peaks^30^. FTIR has a wave number range of 400,000 m^−1^ − 40,000 m^−1^, 32 scans, and a resolution of 400 m^−1^. XPS uses X-rays to measure the kinetic energy of electrons on a material’s surface to calculate the material’s elemental composition and chemical bond formation^31^. In this experiment, monochromatic Al Kα rays (15,000 V, 150 W, hν = 1486.6 eV) were used for determination, and C1s binding energy (284.6 eV) was used as the standard for energy correction. The surface wettability of the material is measured by the water contact angle. The water contact angle measures the surface wettability of a material by measuring the solid-liquid angle of the liquid on the surface of the material^32^. The water contact angle was measured at room temperature, 5 times per sample. The microstructure of the materials was detected by scanning electron microscopy (SEM). SEM scans the sample by focusing a high-energy electron beam and imaging information to obtain the microscopic morphology of the sample^33^. The acceleration voltage used in this experiment is 30,000 V.

### Experimental study on biological properties of PEEK composites in vitro

To evaluate the in vitro biological behavior of MC3T3-E1 osteoblasts on PEEK composite surface, including cell adhesion, proliferation, and differentiation. PEEK samples were placed into 6-well plates and sterilized with ultraviolet light for 1 h. MC3T3-E1 osteoblasts were inoculated on the surface of PEEK material, the adhesion number of cells and the spread of skeleton were observed by SEM, the osteoblastic differentiation ability of MC3T3-E1 cells was evaluated by alkaline phosphatase (ALP) activity detection, and the proliferation of MC3T3-E1 cells was evaluated by CCK-8 detection. The osteogenic activity of the material was evaluated by 1.5 times SBF simulated body fluid immersion in vitro. The SBF solution simulates the ionic components contained in human plasma and can be used to determine whether the material has biological activity in vitro. If the implant material forms a bone-like apatite precipitate in SBF solution, it indicates that the material has osteogenic activity^34^. When evaluating the antibacterial properties of the materials, Staphylococcus aureus and Escherichia coli were cultured in LB and BHI Petri dishes to the logarithmic growth stage, respectively, and 1 × 10^6^CFU mL^−1^ bacterial solution was prepared for use. After being cultured in bacterial solution for 24 h, the material was washed with PBS and ultrasound in a centrifuge tube containing 5 ml sterile PBS buffer for 10 min. The shaken bacterial solution was diluted, 100 µl of diluted suspended bacterial solution was evenly coated in LB/BHI medium, and the number of colony formation (CFU) was calculated after 24 h.

### Study on in vivo repair of rabbit tibial osteonecrosis by PEEK composites

The rabbit tibial osteonecrosis model was established by microwave heating method which has been mature in references^35^. Anesthesia was administered by intraperitoneal injection of pentobarbital sodium 25–30 mg kg^−1^. After successful anesthesia, a longitudinal incision was made on the medial side of the knee joint, about 2 cm long, revealing the tibial plateau. The antenna of the therapeutic instrument was placed in the center of the tibial plateau, the temperature was set at 55℃, and the tibial plateau was inactivated by microwave for 10 min. After inactivation, the incision was sutured with stitches successively. 400,000 units of penicillin were injected into the muscle for three consecutive days after the operation to prevent infection. After 8 weeks of modeling, the medial scar of the knee joint was incised to reveal the tibial plateau. An electric drill with a diameter of 2 mm Kirscher needle was used to drill holes vertically in the center of the tibial plateau and then rinsed with normal saline. According to the random number table method, we randomly divided the thirt-six New Zealand white rabbits into the following six groups (*n* = 6/group): (1)modeled group, (2)PEEK group, (3)SPEEK group, (4)SPEEK-PDA group, (5)SPEEK-PDA-BFP group, and(6)SPEEK-PDA-VEGF group. Meanwhile, we were unaware of the group allocation at the different stages of the experiment (during the allocation, the conduct of the experiment, the outcome assessment, and the data analysis). The rabbits were fed in random cages to minimise potential confounders. No death or lethargy were found in the experimental animals, and all the experimental animals were eventually included in the study. Appropriate anchoring materials were implanted at the drilling site according to the preoperative group. The nail tail was flush with the tibial plateau, and the surgical incision was sutured layer by layer and disinfected with an iodor. Intramuscular injection of 400,000 units of penicillin for three days after surgery to prevent incision infection. The animals were euthanized 12 weeks after surgery by intraperitoneal injection of excess pentobarbital sodium. After the animals were euthanized, the bone tissue samples of the proximal tibia of the affected side were observed by scanning electron microscopy and micro-CT scan. After decalcification, the specimens were divided into two parts after cross-cutting one by one. One sample was dehydrated and transparent, embedded in paraffin, sliced, and then stained by HE. The other slices were cut into 10–15 micron thick slices by OCT embedding and then stained by immunofluorescence. The expressions of angiogenic protein CD31 and osteoblast protein OCN were observed under a laser confocal microscope.

### Statistical methods

The data were processed using SPSS 22.0 statistical analysis software, and it was verified that the data in each group met the requirements of normal distribution. The experimental results in each group were expressed as X ± s (mean ± standard deviation). Intergroup comparisons were made using t-tests, with *P* < 0.05 indicating a statistically significant difference.

## Results

### Preparation and evaluation experiment of PEEK composite materials

**Fig. 2:**
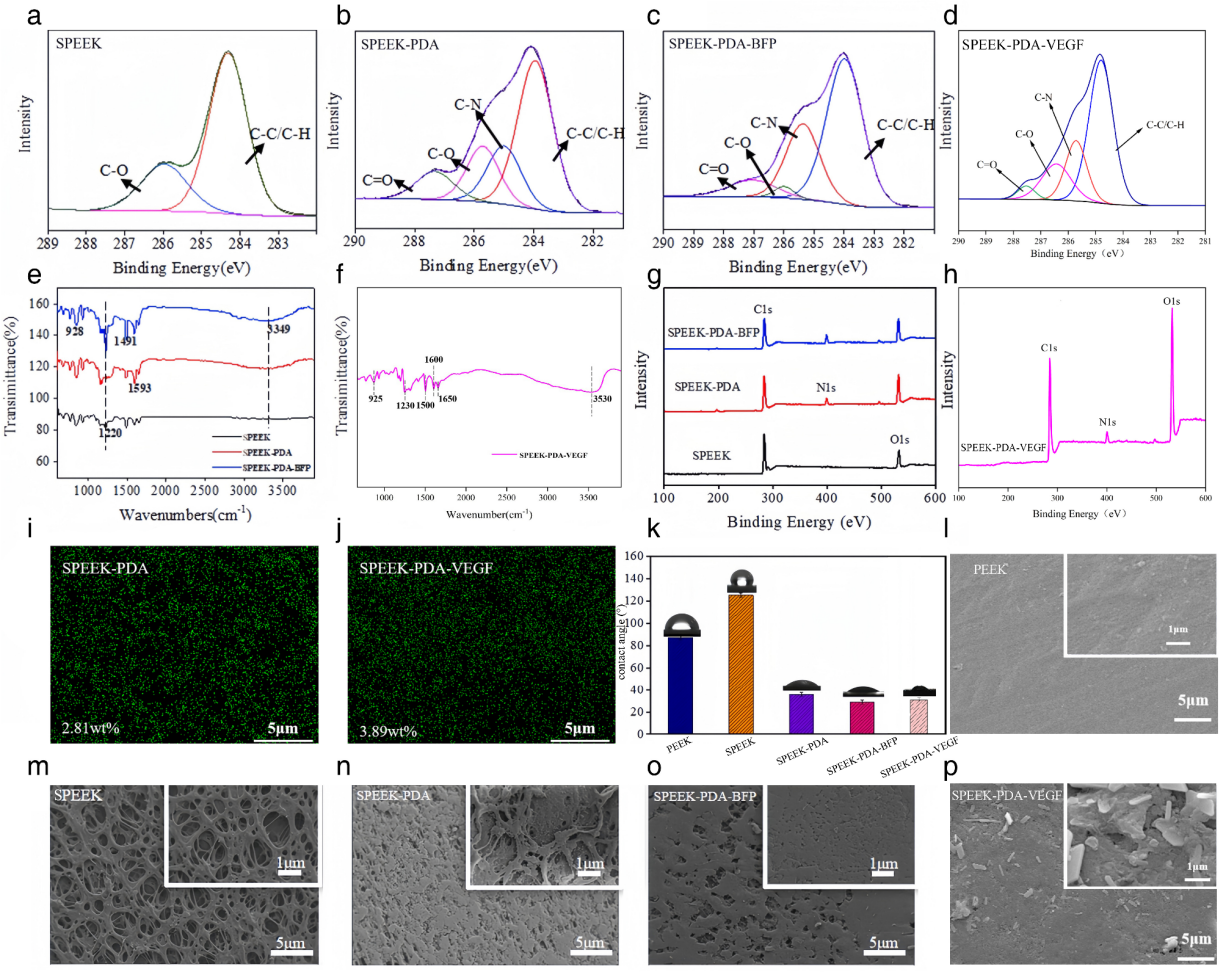
(a) C1s high-resolution spectrogram of SPEEK materials. (b) C1s high-resolution spectrogram of SPEEK-PDA materials. (c) C1s high-resolution spectrogram of SPEEK-PDA-BFP materials. (d) C1s high-resolution spectrogram of SPEEK-PDA-VEGF materials. (e) FTIR diagrams of SPEEK, SPEEK-PDA and SPEEK-PDA-BFP. (f) FTIR diagrams of SPEEK-PDA-VEGF. (g) XPS spectra of SPEEK, SPEEK-PDA and SPEEK-PDA-BFP. (h) XPS spectra of SPEEK-PDA-VEGF. (i) EDS diagram of nitrogen in SPEEK-PDA materials. (j) EDS diagram of nitrogen in SPEEK-PDA-VEGF materials. (k) Surface water contact angles for SPEEK, SPEEK-PDA, SPEEK-PDA-BFP and SPEEK-PDA-VEGF. (l) SEM image of PEEK materials. (m) SEM image of SPEEK materials. (n) SEM image of SPEEK-PDA materials. (o) SEM image of SPEEK-PDA-BFP materials. (p) SEM image of SPEEK-PDA-VEGF materials.

The research group used PDA to graft BFP and VEGF onto the surface of PEEK material and realized the covalent binding of PEEK material and active molecules. As shown in Fig. 1, PEEK material was modified into SPEEK-PDA-BFP and SPEEK-PDA-VEGF after concentrated sulfuric acid sulfonation, PDA crosslinking, and grafting of active factors. The bonding mode and content of the elements contained in the material could be observed by the C1s high-resolution spectrogram. As shown in Fig. 2(a-d), SPEEK-PDA had a strong -C = O peak, which indicated the phthaladione structure in the PDA, which meant that the PDA was grafted onto SPEEK. C-N was the characteristic peak of BFP, and the rise of the peak indicated that BFP was grafted to SPEEK-PDA. The SPEEK-PDA-VEGF material not only possesses carbon skeleton (-C-C-/-C-H-) and hydroxyl (-C-OH) peaks with binding energies of 284.8 eV and 286.4 eV present on the surface of SPEEK and a -C-N- peak with a binding energy of 285.8 eV after the reaction with dopamine. In addition, it also has the -C = O peak with a binding energy of 287.3 eV due to the o-benzoquinone structure that appeared in the process of DA auto polymerization to generate PDA, and the -C-N peak on the surface of the material undergoes a significant rise after grafting by VEGF, which verifies the successful grafting by VEGF. The chemical composition of PEEK composite material was analyzed by FTIR. As shown in Fig. 2(e), the peak at 3349 cm^−1^ was the characteristic peak of the DA phenol hydroxyl group, which indicated that DA was bound to the surface of the SPEEK material. The peaks at 928 cm^−1^ and 1593 cm^−1^ were the characteristic peaks of the PDA phthalate functional groups, which meant that DA self-polymerized to produce PDA. The peaks at 1491 cm^−1^ and 1220 cm^−1^ were the nitrogen-characteristic peaks of BFP, which indicated that BFP was grafted onto SPEEK-PDA. Fig. 2(f) shows the IR spectra of SPEEK-PDA-VEGF tested. The peak at 3530 cm^−1^ is due to the stretching vibration of the phenolic hydroxyl group contained in the dopamine, which can prove that the dopamine was successfully encapsulated on the surface of the SPEEK sample. The peaks at 925 cm^−1^ and 1600 cm^−1^ represent the stretching vibration of the carbonyl group (C = O), which is due to the oxidation of the phenolic hydroxyl group in the dopamine to the o-benzoquinone functional group in the presence of oxygen, a product that occurs in the process of the self-polymerization of dopamine to polydopamine. The peak at 1500 cm^−1^ represents the N-H bending vibration peak in VEGF, indicating that VEGF was successfully loaded onto the SPEEK-PDA matrix material. The peaks appearing at 1230 cm^−1^ and 1650 cm^−1^ correspond to the C-N and carboxylate group stretching vibration peaks, which can also indicate the successful grafting of VEGF. The XPS results of PEEK composites were consistent with those of FTIR. As shown in Fig. 2(g), the characteristic nitrogen element of PDA appeared in SPEEK-PDA. In the broad-spectrum plot in Fig. 2(g), the surface of the sulfonated SPEEK material contains a large amount of C elements as well as O elements, where O elements are mainly due to the concentrated sulfuric acid treatment that endows the surface of the material with more abundant oxygen-containing functional groups. After dopamine modification, the characteristic element N of dopamine appeared, which could prove that dopamine was successfully encapsulated on the material. Fig. 2(h) shows the broad-spectrum of SPEEK-PDA-VEGF. After VEGF grafting, compared with the SPEEK-PDA material, the N content on the surface of the SPEEK-PDA-VEGF material further increased, and the reason for the increase is due to the abundant amino groups in VEGF, which also indicates that VEGF has been successfully grafted on the surface of the material side by side. As shown in Fig. 2(i, j), the content of nitrogen in SPEEK-PDA-VEGF increased due to the grafting of BFP. The hydrophilicity of PEEK materials changed after sulfonation, PDA grafting, and BFP grafting. The hydrophilicity of the material could be obtained by measuring the water contact angle on the surface of the material. The water contact angle results of PEEK, SPEEK, SPEEK-PDA, SPEEK-PDA-BFP and SPEEK-PDA-VEGF were shown in Fig. 2(k). The water contact angle of PEEK material was 85°±1.6°. The water contact angle of the SPEEK material increased to 123°±3.7° and the hydrophilicity decreased. The SPEEK-PDA water contact angle was reduced to 35°±2.5°. The SPEEK-PDA-BFP water contact angle was reduced to 27°±2.7°. The SPEEK-PDA-VEGF water contact angle was 30.84°±2.1°.

The surface modification of PEEK composites would affect the microstructure of the materials. As shown in Fig. 2(l-p), a large number of evenly distributed porous network structures could be seen on the surface of SPEEK.

### Experimental study on biological properties of PEEK composites in vitro

**Fig. 3:**
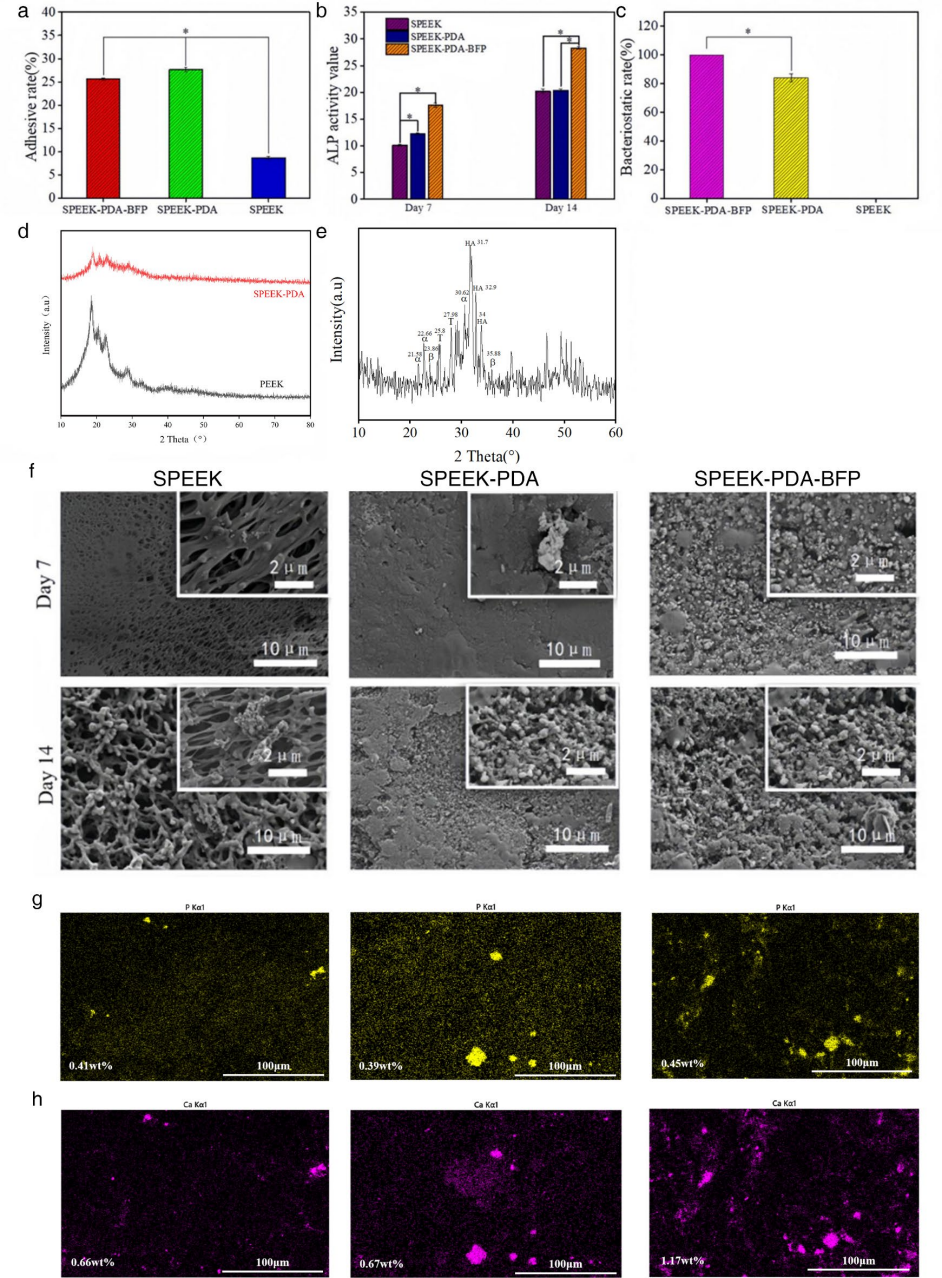
(a) Cell adhesion rates on SPEEK, SPEEK-PDA and SPEEK-PDA-BFP surfaces. (b) ALP activity of SPEEK, SPEEK-PDA and SPEEK-PDA-BFP surface osteoblasts. (c) Bacteriostatic rate of SPEEK, SPEEK-PDA and SPEEK-PDA-BFP surfaces. (d) X-ray emission patterns of PEEK and SPEEK-PDA before immersion. (e) X-ray emission patterns of aggregates on the surface of the material.α is α-TCP, β is β-TCP, T is TTCP, and HA is HA. (f) Mineralization effect of SPEEK, SPEEK-PDA and SPEEK-PDA-BFP in 1.5× SBF solution. (g) EDS diagram of phosphorus in SPEEK, SPEEK-PDA and SPEEK-PDA-BFP materials. (h) EDS diagram of calcium in SPEEK, SPEEK-PDA and SPEEK-PDA-BFP materials.

The ideal orthopedic graft should be conducive to the adhesion proliferation and osteogenic differentiation of osteoblasts^36,37^. As shown in Fig. 3(a), the adhesion of osteoblasts on the SPEEK surface was minimal. This was because the sulfonated surface of SPEEK was an inert surface and therefore had limited adhesion to cells. The cell adhesion of SPEEK-PDA and SPEEK-PDA-BFP was close to and better than SPEEK (*p* < 0.05).

We used alkaline phosphatase activity of osteoblasts to examine the in vitro osteogenic activity of different composites. As shown in Fig. 3(b), on the 7th day of cell culture, the ALP activity of the three materials was significantly increased, with the ALP activity of SPEEK-PDA-BFP remaining higher than that of SPEEK and SPEEK-PDA (*p* < 0.05). On the 14th day, there was no significant difference in the ALP activity of SPEEK and SPEEK-PDA surface cells, whereas the ALP activity of SPEEK-PDA-BFP was significantly higher than that of SPEEK and SPEEK-PDA (*p* < 0.05).

We evaluated the bacteriostasis of the material by measuring the bacteriostasis rate of the material surface. As shown in Fig. 3(c), SPEEK had almost no antibacterial effect, and SPEEK-PDA and SPEEK-PDA-BFP had excellent antibacterial effect, among which SPEEK-PDA-BFP had the best antibacterial effect (*p* < 0.05).

The SEM images of PEEK composite material soaked in 1.5 times SBF solution for 7 days and 14 days were shown in Fig. 3(f). When the material was soaked to the 7th day, almost no precipitation formed on SPEEK and SPEEK-PDA, only small spherical particles appeared, while more aggregates appeared on the surface of SPEEK-PDA-BFP. When the material was soaked on the 14th day, a large number of uniform clumped particles appeared on the surface of SPEEK and SPEEK-PDA. However, the number of SPEEK-PDA-BFP aggregates also increased significantly, and the aggregates were closely bound to the material. As shown in Fig. 3(d), X-ray diffraction was performed on PEEK and SPEEK-PDA before immersion. PEEK showed characteristic XRD peaks at 2θ of 18.98°, 20.79°, 22.83°, and 28.91°, which represent diffraction in the (110), (111), (200), and (211) planes, respectively; SPEEK-PDA The diffraction peaks of SPEEK-PDA became flat and the intensity decreased, which was due to the introduction of the _−SO3H_ group blocking the orderly arrangement of the polymer chain, resulting in the reduction of polymer crystallization, PDA was a steamed bun peak in the XRD test, and no obvious characteristic peaks existed; BFP is a part of the osteoblastically active proteins, and VEGF is a protein structure that exists with eight conserved cysteine residues and one cystine. protein structure, both BFP and VEGF have no specific structure and have no specific characteristic peaks in the XRD test. As shown in Fig. 3(e), X-ray diffraction was performed on the aggregate on the surface of the material. It could be seen that the aggregate was composed of hydroxyapatite (HA), tricalcium α-phosphate (α-TCP), tricalcium β-TCP (β-TCP), and tricalcium phosphate (TCP). As shown in Fig. 3(g-h), the SPEEK material contains 0.41 wt% of P element and 0.66 wt% of Ca element. The SPEEK-PDA material contains 0.39 wt% of P element and 0.67 wt% of Ca element. The SPEEK-PDA-BFP material contains 0.45 wt% of P element and 1.17 wt% of Ca element.

### Study on in vivo repair of rabbit tibial osteonecrosis by PEEK composites

**Fig. 4:**
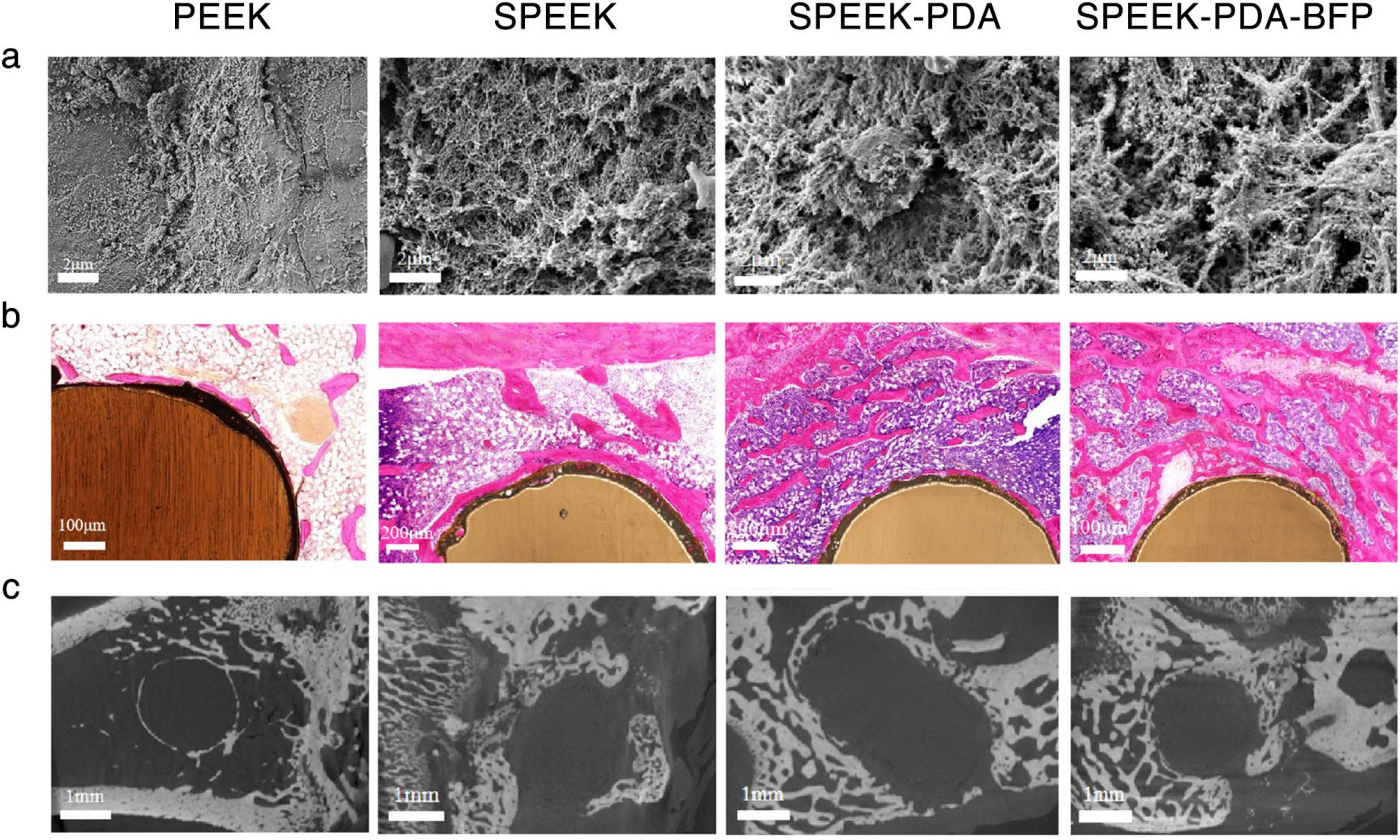
(a) SEM images of bone tissue around PEEK, SPEEK, SPEEK-PDA and SPEEK-PDA-BFP. (b) HE stained sections of bone tissue around PEEK, SPEEK, SPEEK-PDA and SPEEK-PDA-BFP. (c) micro-CT images of bone tissue surrounding PEEK, SPEEK, SPEEK-PDA and SPEEK-PDA-BFP.

In this study, a rabbit osteonecrotic model was established by microwave heating with traumatic modeling, and then PEEK-modified anchors were implanted at the osteonecrotic site to evaluate the bone repair ability of the material in the animals. After 12 weeks of PEEK-modified anchor implantation in rabbit tibia, SEM imaging, HE staining and micro-CT examination were performed on the osteonecrosis area. As shown in Fig. 4(a), there was no obvious bone trabecular formation around PEEK. There were sparse bone trabecular structures on the SPEEK, and the bone maturity was low. SPEEK-PDA had a dense distribution of new bone trabecular formation, and deep into the internal space of SPEEK-PDA. The surface of SPEEK-PDA-BFP was covered with compact bone trabeculae arranged in a honeycomb pattern, and the new bone trabeculae were almost completely fused with the original bone tissue. As shown in Fig. 4(b), no trabecular formation of new bone was observed around PEEK. A small amount of random new bone trabecular tissue was formed at the edge of the bone defect around SPEEK. However, there were still obvious gaps between new bone tissue and SPEEK, and the osteogenic effect of the material was poor. More regular and robust bone trabecular structures appeared around SPEEK-PDA, and the repair degree of bone defects was higher. The gap between SPEEK-PDA and new bone tissue was small, some fibroblasts grew to the surface of SPEEK-PDA, and a large number of osteoblasts and bone marrow cells could be seen. More regular and dense bone trabecular structure could be seen around SPEEK-PDA-BFP, and part of bone tissue covered the surface of SPEEK-PDA-BFP and grew along the boundary. SPEEK-PDA-BFP formed a dense connection with the new bone trabecula. There was a large area of bone tissue and neovascularization around SPEEK-PDA-BFP. As shown in Fig. 4(c), no new bone tissue formation was observed around the bone defect area of PEEK, and the bone repair effect was not obvious. New bone tissue was formed around the bone defect of SPEEK and SPEEK-PDA, and the repair area of the bone defect was increased. The bone defect of SPEEK-PDA-BFP was surrounded by continuous and dense bone trabecular structures. In conclusion, SPEEK-PDA-BFP had the best bone defect repair ability.

### Comparative study of SPEEK-PDA-VEGF and SPEEK-PDA-BFP in the treatment of osteonecrosis

Through the above experiments, we verified SPEEK-PDA-BFP’s excellent bone repair and bone integration capabilities. Then, we further investigated the ability of SPEEK-PDA-VEGF and SPEEK-PDA-BFP PEEK composites to repair osteonecrosis.

**Fig. 5:**
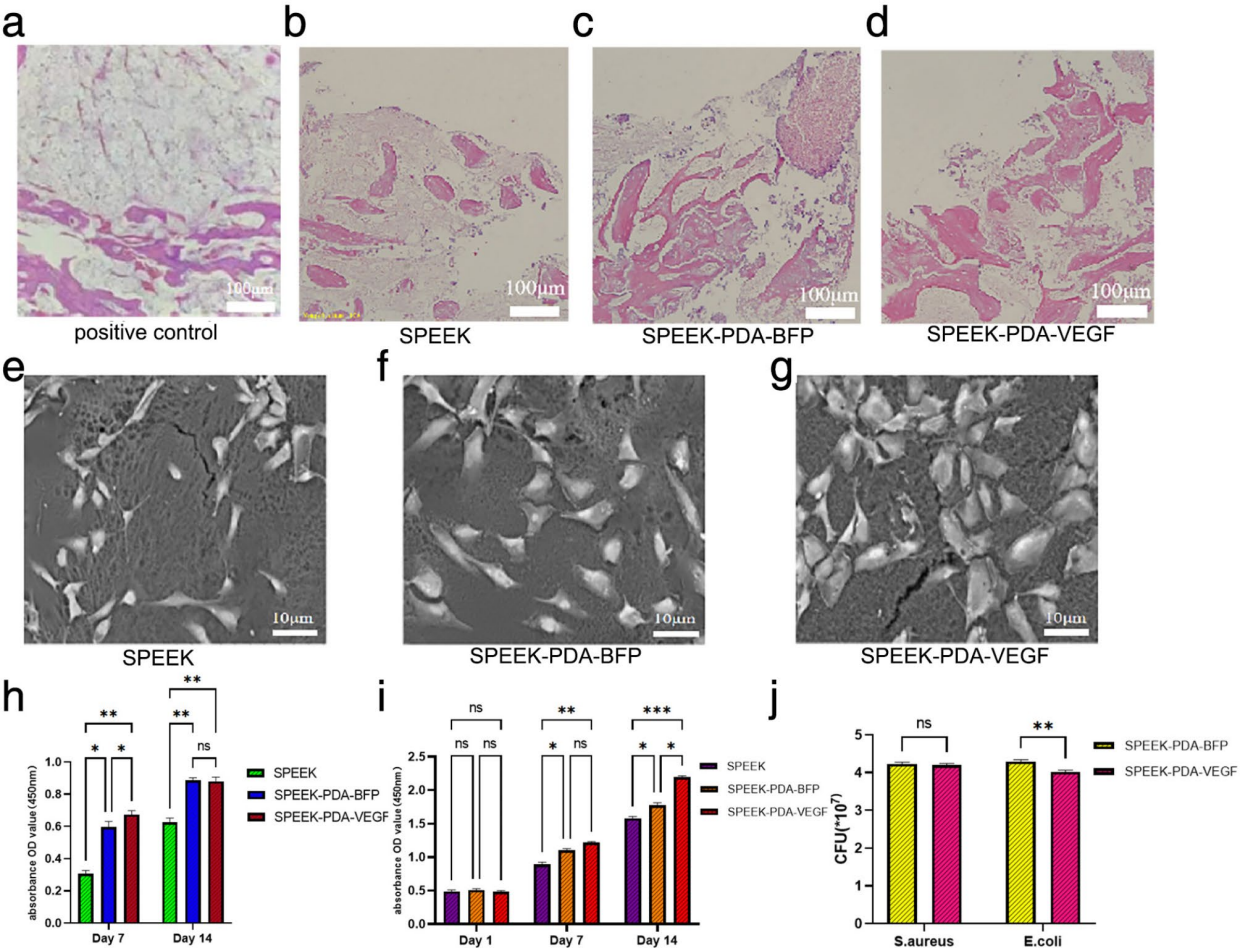
(a) HE stained section of osteonecrosis. (b) HE stained sections of bone tissue around SPEEK. (c) HE stained sections of bone tissue around SPEEK-PDA-BFP. (d) HE stained sections of bone tissue around SPEEK-PDA-VEGF. (e) Adhesion of osteoblasts on the surface of SPEEK. (f) Adhesion of osteoblasts on the on the surface of SPEEK-PDA-BFP. (g) Adhesion of osteoblasts on the surface of SPEEK-PDA-VEGF. (h) The effect of SPEEK, SPEEK-PDA-BFP and SPEEK-PDA-VEGF on osteoblast differentiation. (i) The effect of SPEEK, SPEEK-PDA-BFP and SPEEK-PDA-VEGF on osteoblast proliferation. (j) CFU quantitative analysis of SPEEK-PDA-BFP and SPEEK-PDA-VEGF surface bacteria.

HE staining of bone tissue around the material was shown in Fig. 5(a-d). As could be seen from the positive control in Fig. 5(a), bone marrow necrosis and empty bone lacunae of bone trabeculae were observed in osteonecrotic tissue without PEEK composite material implantation. As shown in Fig. 5(b-d), only a small amount of sparsely scattered new bone tissue was generated around SPEEK. There were more regular and dense bone trabeculae around SPEEK-PDA-BFP and more new bone cells. The osteonecrotic area around SPEEK-PDA-VEGF was replaced by a large number of mature and dense bone trabeculae, and the new osteocytes differentiated and matured.

We used SEM to detect the adhesion of osteoblasts on the surface of the material. As shown in Fig. 5(e-f), after osteoblast culture for 24 h, the number of cell adhesions on the surface of SPEEK was less and the volume was smaller. The number of adherent cells on the surface of SPEEK-PDA-VEGF and SPEEK-PDA-BFP increased significantly, and the cell volume increased, especially the number of adherent cells on the surface of SPEEK-PDA-VEGF was the highest.

ALP activity assay was used to evaluate the differentiation ability of osteoblasts, and absorbance OD values were obtained, as shown in Fig. 5(h). On the 7th and 14th day of cell culture, the OD values of SPEEK, SPEEK-PDA-BFP, and SPEEK-PDA-VEGF showed an increasing trend. At the same time point, SPEEK-PDA-BFP and SPEEK-PDA-VEGF showed higher ALP activity (*P* < 0.05). On the 7th day, SPEEK-PDA-VEGF showed higher ALP activity compared to SPEEK-PDA-BFP. On the 14th day, there was no significant difference between the two (*P* > 0.05). We evaluated the proliferation capacity of osteoblasts by CCK-8 assay. As shown in Fig. 5(i), OD values of the three materials showed an increasing trend on the 1st, 4th, and 7th day of cell culture. On the 1st day, the OD values of the three materials were not significantly different (*P* > 0.05). On the 4th day, the OD values of SPEEK-PDA-BFP and SPEEK-PDA-VEGF were higher than those of SPEEK (*P* < 0.05), but the OD values of SPEEK-PDA-BFP and SPEEK-PDA-VEGF were not significantly different (*P* > 0.05). On the 7th day, the OD values of SPEEk-PDA-BFP and SPEEK-PDA-VEGF were higher than SPEEK, and the OD values of SPEEK-PDA-VEGF were higher than SPEEK-PDA-BFP (*P* < 0.05). In conclusion, SPEEK-PDA-BFP and SPEEK-PDA-VEGF promoted the proliferation and differentiation of osteoblasts, and SPEEK-PDA-VEGF in particular had an excellent osteogenic ability.

We tested the bacteriostatic activity of SPEEK-PDA-BFP and SPEEK-PDA-VEGF against Staphylococcus aureus (S.aureus) and Escherichia coli (E.coli). As shown in Fig. 5(j), the adhesion number of E. coli on the surface of SPEEK-PDA-VEGF was less than that of SPEEK-PDA-BFP (*P* < 0.05), while the adhesion number of S.aureus on the surface of SPEEK-PDA-BFP and SPEEK-PDA-VEGF was not significantly different (*P* > 0.05).

**Fig. 6:**
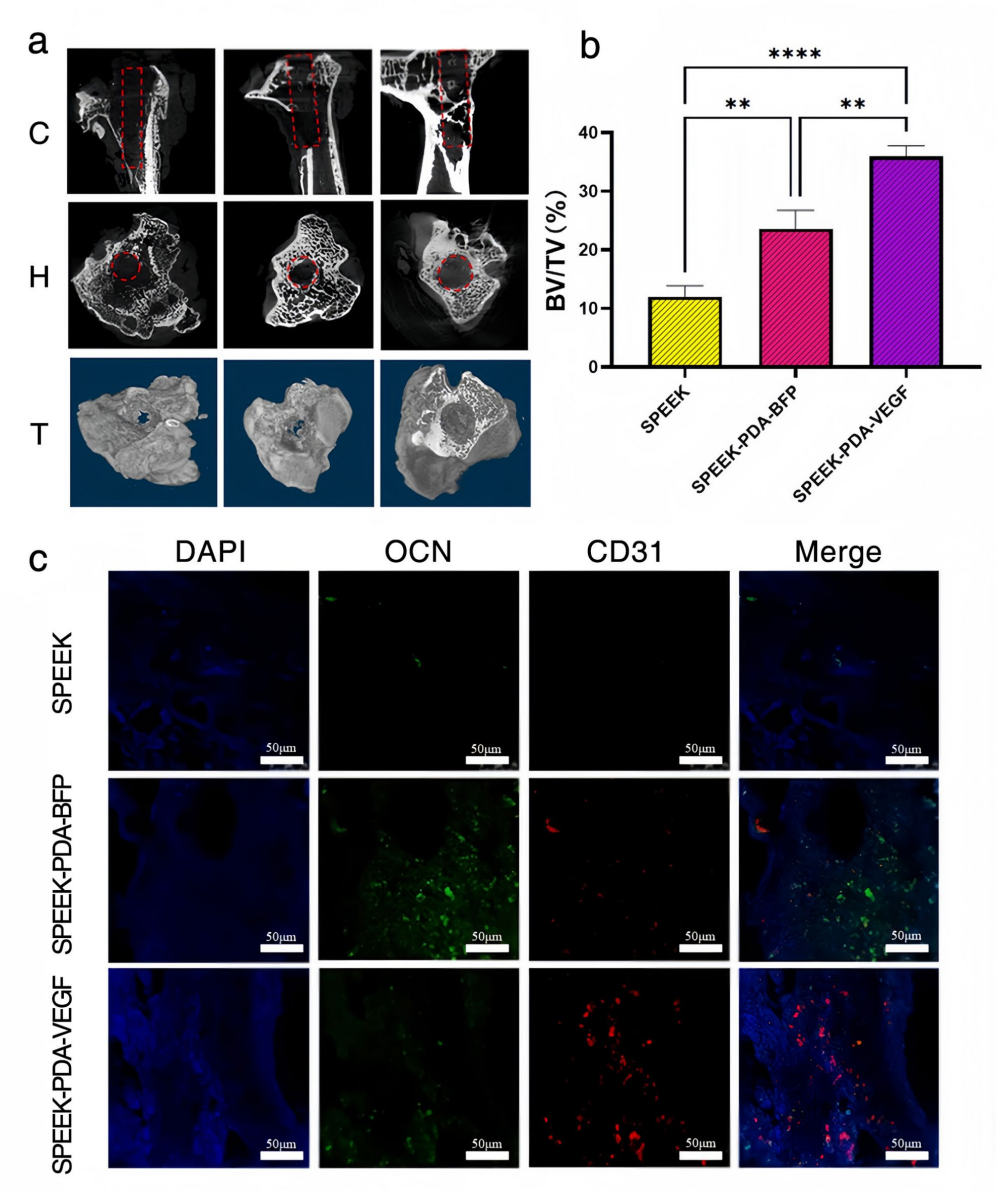
(a) micro-CT images of bone tissue surrounding SPEEK, SPEEK-PDA-BFP, and SPEEK-PDA-VEGF.C is the coronal plane, H is the horizontal plane, and T is the three-dimensional reconstruction. (b) BV/TV values of bone tissue around SPEEK, SPEEk-PDA-BFP and SPEEK-PDA-VEGF.(c) Immunofluorescence staining of bone tissue around SPEEK, SPEEK-PDA-BFP, and SPEEK-PDA-VEGF.

We used micro-CT to detect bone reconstructions around SPEEK, SPEEK-PDA-BFP, and SPEEK-PDA-VEGF. As shown in Fig. 6(a), little and sparse bone tissue was generated around SPEEK. A large number of robust new bone trabeculae were formed around SPEEK-PDA-BFP, and the area of dead bone and bone defect was significantly reduced. The bone trabeculae around SPEEK-PDA-VEGF were dense and thick, no obvious bone defects were found, and the new bone filling was the most obvious. We quantitatively analyzed the micro-CT images and obtained the BV/TV values of the new bone tissue around osteonecrosis. As shown in Fig. 6(b), the BV/TV value of bone tissue around SPEEk-PDA-BFP was higher than that of SPEEK (*P* < 0.05), and the BV/TV value of bone tissue around SPEEK-PDA-BFP was higher than that of SPEEK-PDA-BFP (*P* < 0.05). This indicated that SPEEK-PDA-BFP and SPEEK-PDA-VEGF both effectively promoted the repair of necrotic bone tissue, and SPEEK-PDA-VEGF had the better osteogenic ability.

The bone tissue around the composite was stained by immunofluorescence. As shown in Fig. 6(c), the expressions of CD31 angiogenic protein and OCN osteoblast protein in the surrounding tissue of SPEEK were lower. OCN osteogenic protein was highly expressed in the surrounding tissues of SPEEK-PDA-BFP, while CD31 angiogenic protein was hardly expressed. High expression of CD31 angiogenic protein and small expression of OCN osteoblastic protein were observed in the surrounding tissues of SPEEK-PDA-VEGF.

## Discussion

### Advantages of hydrophilicity and microstructure of PEEK composite materials

The enhanced hydrophilicity and surface energy facilitated cell adhesion and proliferation. The water contact angle of PEEK material was 85°±1.6°, which meant that PEEK material that had not been sulfonated was close to a hydrophobic state. The water contact angle of the SPEEK material increased to 123°±3.7° and the hydrophilicity decreased. We speculated that this was due to the large number of tiny pore structures generated on the surface of the SPEEK material, which reduced the free energy and hydrophilicity of the material surface. The SPEEK-PDA water contact angle was reduced to 35°±2.5°. This was due to the fact that PDA provided groups such as phenol hydroxyl and phthalate, which increased the free energy and wettability of the material. The SPEEK-PDA-BFP water contact angle was reduced to 27°±2.7°. The increased hydrophilicity and surface energy were conducive to cell adhesion and proliferation on SPEEK-PDA-BFP surface. The SPEEK-PDA-VEGF water contact angle was 30.84°±2.1°. Compared with the contact angle of SPEEK-PDA of 35°±3.5°, the hydrophilicity of the surface of the SPEEK-PDA-VEGF material was enhanced, which was attributed to the presence of hydrophilic group carboxyl groups on VEGF, and when the amino group of VEGF reacted with the phenolic hydroxyl group of SPEEK-PDA in a Michael addition reaction, hydrophilic group carboxyl groups on the structure of VEGF were grafted together to form SPEEK -When the amino group of VEGF and the phenolic hydroxyl group of SPEEK-PDA undergo Michael addition reaction, the carboxyl group of the hydrophilic group on the structure of VEGF is grafted together to form SPEEK-PDA-VEGF, which results in the decrease of contact angle and increase of hydrophilicity.

The surface modification of PEEK composites would provided sites for the grafting of active factors. The hole structure on the surface of SPEEK-PDA was coated by PDA, forming a uniform PDA film. This layer of PDA film provided sites for the grafting of active factors such as BFP. A large number of small particles could be seen in the pore structure of SPEEK-PDA-BFP, and these particles were the BFP grafted on the surface of SPEEK-PDA. The surface of SPEEK-PDA-VEGF is covered with many homogeneous micron-sized flakes or rods. These flakes or rods are the chemically grafted VEGF.

### PEEK composites enhance the adhesion, proliferation, and osteogenic differentiation of osteoblasts

The cell adhesion of SPEEK-PDA and SPEEK-PDA-BFP was close to and better than SPEEK (*p* < 0.05). The above results showed that the surface energy and hydrophilicity of SPEEK increased after PDA coating and BFP grafting, which promoted the proliferation and adhesion of osteoblasts. The results of the cell adhesion experiment were consistent with those of the water contact angle experiment.

The results of alkaline phosphatase activity of osteoblasts indicated that PEEK-PDA-BFP could significantly promote the proliferation and differentiation of osteoblasts under the synergistic effect of PDA and BFP.

The bacteriostasis rate of the material surface showed that SPEEK-PDA-BFP had the best antibacterial effect. We speculated that this was mainly due to the fact that PDA and BFP could inhibit the growth of bacteria^38,39^, and the synergistic effect of PDA and BFP made SPEEK-PDA-BFP achieve the optimal antibacterial effect.

Differences in aggregate formation at different times indicated that SPEEK-PDA-BFP could more effectively trigger the formation of bone-like apatite precipitates and had more excellent osteogenic activity.

### PEEK composites exhibit remarkable capabilities in repairing bone defects

Through the experimental study in vitro, we confirmed that the PEEK composite had good biocompatibility and bone induction ability, but compared with a single culture environment in vitro, the influencing factors in vivo were more complex. The blood supply of the osteonecrosis site was seriously damaged, which was not conducive to the proliferation and differentiation of osteoblasts, and bone regeneration was more difficult^40^. We set up a rabbit osteonecrosis model by microwave heating to verify the repair ability of the defect in vivo. Compared with the osteonecrosis modeling method of hormone injection, microwave heating modeling could accurately regulate the necrotic site, simple operation, high success rate, and strong repeatability. Although microwave heat injury was difficult to occur clinically, the pathological manifestations of bone tissue injury were very similar to osteonecrosis. SEM imaging showed that the SPEEK revealed sparse trabecular bone structures, signifying a low level of bone maturity. Due to the low hydrophilicity and surface energy of SPEEK, the bone trabecular structure was only attached to the surface of SPEEK, and did not extend into the internal space of SPEEK, which maked it difficult for the new bone tissue to bind closely with SPEEK. The osteogenic activity of SPEEK-PDA was improved by the improvement of hydrophilicity and surface properties. SPEEK-PDA-BFP’s superior osteogenic activity confirmed the bone-promoting ability of BFP. HE staining revealed that a large number of osteoblasts and bone marrow cells were present on the surface of SPEEK-PDA, indicating better biocompatibility. There was a large area of bone tissue and neovascularization around SPEEK-PDA-BFP. which indicated that BFP could promote both osteogenesis and angiogenesis, promote bone tissue growth and blood revascularization. Micro-CT analysis revealed that SPEEK-PDA-BFP exhibited the best bone defect repair capability.

All in all, SPEEK-PDA-BFP demonstrated superior bone defect repair efficacy. Among them, PEEK connected and supported new bone tissue in the process of bone reconstruction, PDA promoted the adhesion and proliferation of osteoblasts, and BFP promoted the formation and differentiation of bone tissue.

### SPEEK-PDA-VEGF showed better bone repair effects in the comparative study

Studies^41,42^ have shown that VEGF plays an important role in the process of bone repair. VEGF could promote the proliferation and differentiation of vascular endothelial cells and induce angiogenesis at the site of osteonecrosis. VEGF could promote the proliferation and differentiation of osteoblasts and participate in the regeneration and repair of bone tissue. Compared with BFP, which only had osteogenic activity, VEGF with both osteogenic and angiogenic activity could significantly improve the bone integration of PEEK materials, improve blood supply disorders at osteonecrosis sites, and reverse osteonecrosis. Therefore, we conducted a comparative study of SPEEK-PDA-VEGF and SPEEK-PDA-BFP PEEK.

In HE staining, around SPEEK-PDA-BFP, there were more regular and dense bone trabeculae, along with a greater number of newly formed bone cells. The area of bone necrosis around SPEEK-PDA-VEGF was replaced by a large amount of mature and dense bone trabeculae, with the new bone cells undergoing differentiation and maturation. According to research reports^43,44^, VEGF is the most powerful growth factor in promoting vascular regeneration. VEGF accelerated the regeneration and repair of bone tissue through the bidirectional regulation of osteogenesis and angiogenesis, and provided sufficient blood supply for bone repair, endowing the material with more excellent osteogenic effects. During bone growth, significant up-regulation of local VEGF concentration was conducive to the formation of neovascularization and the mineralization of chondrocytes around blood vessel growth. However, the content of VEGF was low in the osteonecrosis area, so the introduction of exogenous VEGF was more important. The introduction of exogenous VEGF up-regulated the expression of endogenous VEGF, promoted vascular reconstruction in the bone defect area, and accelerated the formation of new bone^45^.

We observed that the surface of SPEEK-PDA-VEGF had the highest number of adherent cells, as determined by SEM. We believed that this was mainly due to the formation of rough and dense pore structures on the surface of SPEEK material, which increased the surface area of the material and facilitated the adhesion and anchoring of cells. The grafting of VEGF, BFP, and other growth factors further promoted the chemical binding of materials and cells. This suggested that the two modification methods of material sulfonation treatment and growth factor coating modification could play a complementary superposition effect, and further improve the biological activity of the material. Among various composites, SPEEK-PDA-VEGF showed the best cell adhesion. We speculated that this may be due to VEGF having a larger protein volume. The larger protein volume allowed SPEEK-PDA-VEGF to have a rougher surface, which was conducive to osteoblast adhesion and proliferation.

We evaluated the differentiation ability of osteoblasts using ALP activity assay, and concluded that SPEEK-PDA-BFP and SPEEK-PDA-VEGF promoted the proliferation and differentiation of osteoblasts, especially SPEEK-PDA-VEGF had better osteogenic ability.

The bacteriostasis experiment showed that SPEEK-PDA-VEGF had higher bacteriostasis rate for Escherichia coli than SPEEK-PDA-BFP, but the difference was not obvious for Staphylococcus aureus. However, studies^46,47^ have shown that VEGF could up-regulate the expression of a variety of immune proteins, strengthen the adhesion, chemotaxis, and phagocytosis functions of macrophages, and then kill bacteria. Therefore, the antibacterial properties of SPEEK composites may not be exactly the same in vitro and in vivo, which was the direction we need to further study.

Micro-CT test results showed that SPEEK-PDA-BFP and SPEEK-PDA-VEGF both effectively promoted the repair of necrotic bone tissue, and SPEEK-PDA-VEGF had better bone promoting ability.

Under immunofluorescence staining, we observed that the expression of CD31 angiogenic protein and OCN osteogenic protein was lower in the tissue surrounding SPEEK. In the tissue surrounding SPEEK-PDA-BFP, a high expression of OCN osteogenic protein was visible, while the expression of CD31 angiogenic protein was almost absent. In the tissue surrounding SPEEK-PDA-VEGF, a high expression of CD31 angiogenic protein and a low expression of OCN osteogenic protein were observed. This indicates that the implantation of exogenous VEGF not only promoted the expression of endogenous VEGF but also enhanced the expression of endogenous BFP.

### Key research highlights and future prospects

We have reviewed and summarized the latest studies on PEEK materials, comparing them with our own study. We have identified three main highlights in our study:

A novel design of modified materials using BFP and VEGF as grafted molecules. Previous studies have achieved material modification through various methods, such as varying the degree of sulfonation^48^, using laser polishing technology^49^, or combining with different substances like ions and bioactive materials^50,51^. Research that incorporates bioactive materials, especially using BFP and VEGF, is relatively scarce, which underscores the novelty of our approach.

Comprehensive in vitro and in vivo experiments that enhance the scientific rigor and credibility of the study. Some studies have only conducted in vitro cell or antibacterial experiments^48–50,52,53^, while others have focused solely on in vivo experiments^51^. There are also studies that have investigated both in vitro and in vivo aspects^54–56^. Our study has adopted both in vitro and in vivo experiments, further solidifying the scientific validity and persuasiveness of our findings.

Promising future applications and research prospects. Our research addresses the widespread clinical issue of osteonecrosis, and the modified materials have the potential to be applied in clinical settings. This is undoubtedly crucial for the benefit of patients with osteonecrosis. Additionally, we have found that certain studies have delved into the mechanisms of action, which involve modulating immune responses^50,51,55^. One study has discovered that the modified materials may function through a specific pathway, such as the Akt/GSK-3β/β-catenin signaling pathway, exerting certain effects and thereby achieving its goals^54^. These findings suggest that investigating the mechanisms by which our novel modified materials, grafted by BFP and VEGF, function is important and that the research potential in this direction is significant.

## Conclusion

In this study, we developed a new PEEK composite material and studied the effect of this PEEK material on osteonecrosis. Through concentrated sulfuric acid sulfonation, polydopamine coating, and grafting of growth factors, we solved the problems of biological inertia, low surface energy, and poor biological activity of PEEK materials, and finally constructed a PEEK composite material with excellent biocompatibility and bone integration ability. The sulfonation of concentrated sulfuric acid resulted in the appearance of a micrometer-level three-dimensional pore structure on the surface of PEEK materials, which provided sites for cell and protein adhesion. As a crosslinking agent, PDA grafted growth factors with osteogenic activity such as BFP and VEGF in the form of chemical binding, which increased the osteogenic activity of cells and improved the osteogenic properties of materials. Among the PEEK composite materials, SPEEK-PDA-VEGF composites had the best bone repair effect, which may be related to the strong osteogenic and angiogenic activities of VEGF. Therefore, we can conclude that PEEK composites are an effective strategy for the treatment of osteonecrosis.

## Data Availability

The raw data required to reproduce these findings are available to download from [Sun, Yi; Ren, Qiang (2024), “Polydopamine Grafting Polyether Ether Ketone to Stabilize Growth Factor for Efficient Osteonecrosis Repair”, Mendeley Data, V1, doi: 10.17632/8drj65w9g7.1]. The processed data required to reproduce these findings are available to download from [Sun, Yi; Ren, Qiang (2024), “Polydopamine Grafting Polyether Ether Ketone to Stabilize Growth Factor for Efficient Osteonecrosis Repair”, Mendeley Data, V1, doi: 10.17632/8drj65w9g7.1].
